# PinMyMetal: A hybrid learning system to accurately model metal binding sites in macromolecules

**DOI:** 10.21203/rs.3.rs-3908734/v1

**Published:** 2024-02-21

**Authors:** Heping Zheng, Huihui Zhang, Juanhong Zhong, Michal Gucwa, Yishuai Zhang, Haojie Ma, Lei Deng, Longfei Mao, Wladek Minor, Nasui Wang

**Affiliations:** Hunan University College of Biology; Hunan University College of Biology; Hunan University College of Biology; Jagiellonian University; Hunan University College of Biology; Hunan University College of Biology; Hunan University College of Biology; Hunan University College of Biology; University of Virginia; Division of Endocrinology and Metabolism, The First Affiliated Hospital of Shantou University Medical College

## Abstract

Metal ions are vital components in many proteins for the inference and engineering of protein function, with coordination complexity linked to structural (4-residue predominate), catalytic (3-residue predominate), or regulatory (2-residue predominate) roles. Computational tools for modeling metal ions in protein structures, especially for transient, reversible, and concentration-dependent regulatory sites, remain immature. We present PinMyMetal (PMM), a sophisticated hybrid machine learning system for predicting zinc ion localization and environment in macromolecular structures. Compared to other predictors, PMM excels in predicting regulatory sites (median deviation of 0.34 Å), demonstrating superior accuracy in locating catalytic sites (median deviation of 0.27 Å) and structural sites (median deviation of 0.14 Å). PMM assigns a certainty score to each predicted site based on local structural and physicochemical features independent of homolog presence. Interactive validation through our server, CheckMyMetal, expands PMM’s scope, enabling it to pinpoint and validates diverse functional zinc sites from different structure sources (predicted structures, cryo-EM and crystallography). This facilitates residue-wise assessment and robust metal binding site design. The lightweight PMM system demands minimal computing resources and is available at https://PMM.biocloud.top. While currently trained on zinc, the PMM workflow can easily adapt to other metals through expanded training data.

## 1. Introduction

Metal ions play a crucial role in the structure and function of macromolecules^1^, acting as essential cofactors for many enzymes and influencing various molecular and cellular processes^2^. About one-third of proteins in known genomes require metal ions to maintain their natural structure and function. However, only a small fraction of metal binding proteins have been elucidated^3, 4^. Understanding the location of metals in proteins and their interactions is essential for designing new drug synthesis pathways and modifying biological functions^5, 6, 7, 8^. For example, the most abundant metal ion in the Protein Data Bank (PDB) is zinc, which is crucial in diseases, drug targeting, stability, and regulation^9^.

Metal-protein complexes studies benefit from experimental methods, yet face artifacts like incorrect metal incorporation and ion removal during purification^10^. In addition, experimental methods face resolution limitations when determining metal binding structures, particularly in cryo-electron microscopy (cryo-EM). Despite the success of cryo-EM in large and complex macromolecules, electron penetration depth and scattering effects hinder high-resolution imaging of metal ions^11^. Computational predictions offer advantages including cost-effectiveness, scalability, and high-throughput. Combining both approaches provides a more comprehensive understanding of metal sites in proteins. Metal sites typically comprise amino acids close in 3D structure but distant in sequence, posing a challenge to identify sites with short amino acid spacers between ligands, such as regulatory sites^12^. Hence, structure-based predictions are expected to outperform sequence-based methods^13, 14, 15^. Advancements in protein structure prediction, exemplified by Alphafold2, show promise for accurate predictions of protein structures, offering opportunities and challenges in annotating metal sites in computational models^16^.

Existing structure-based metal site predictors employ diverse approaches. BioMetAll^5^, TEMSP^17^, and GRE4Zn^13^ use geometric features, such as metal-ligand distances. CHED^18^ focuses on triads of metal-coordinating ligand residues in apoprotein structures. ZincBindDB classifies zinc sites into ten classes, employing machine learning models based on structural characteristics^19^. MIB^20, 21^, and AlphaFill^22^ infer the presence of metal ions based on homology to known metal binding structures. Metal3D employs a deep learning algorithm with a voxelized protein environment representation^23^.

These predictors can be divided into three categories: (I) binding site predictors for metal binding residues (CHED^18^, ZincBindDB^19^); (II) binding position predictors for metal ion coordinates (Metal3D^23^, BioMetAll^5^, AlphaFill^22^); (III) predictors that identify both residues and coordinates (TEMSP^17^, GRE4Zn^13^, MIB^20, 21^). However, these methods have significant drawbacks. BioMetAll lacks templates and a confidence metric but provides many potential binding site locations on a grid, whose strategy finds the site at the cost of increasing site uncertainty^5^. CHED, TEMSP, GRE4Zn, and MIB exclude metal sites with two or fewer coordinating ligands. Metal3D can only predict the coordinates of metal ions and has a long prediction time, unsuitable for large-scale predictions^23^. Homology-based predictors like MIB, AlphaFill, and ZincBindDB can successfully find sites that match known metal site patterns, while identifying metal binding sites in proteins lacking sufficient homologous structural domains or motifs remains challenging. The structure-based hybrid machine learning system developed herein, named PinMyMetal (PMM), overcomes these drawbacks to predict both metal location and coordinating ligands.

Metal ions in proteins are typically coordinated by Cysteine(C), Histidine(H), Glutamate(E), and Aspartate(D)^24^, with sulfur and nitrogen donors increasing site stability according to hard and soft acids and bases principles^25^, specifically C and H ligands^26, 27^. While alkali and alkaline earth metals tend to commonly serve structural roles, transition metals are more versatile in function, as exemplified by the most abundant transition metal zinc in PMM. The varied functions of zinc binding sites exhibit distinct structures^14, 28^. Functionally, these sites are generally divided into structural, catalytic, and regulatory sites, predominantly coordinated by four, three, and two residues, respectively^29, 30, 31^. The PMM system uses C and H residues as the primary measure and ED as an auxiliary measure. It categorizes binding sites by functionality into three groups, employing different optimal strategies for each functional group to formulate a hybrid machine learning approach to predict zinc binding sites. Trained on 20,979 non-redundant high-quality zinc binding sites validated by CheckMyMetal (CMM)^32, 33^, the PMM system incorporates predicted sites into protein structures and further validates them using CMM. It efficiently screens and validates both metal ion locations and coordinating ligands throughout the protein based on amino acid type, location coordinates, structural characteristics, and surrounding hydrophilic profile.

While the current workflow of PMM is trained using zinc binding sites, it also gives informative cues about other common transition metal binding sites (Mn, Fe, Co, Ni, Cu, Cd). Yet, the modeling of non-zinc metal ions should be interpreted carefully. The underlying workflow of PMM is readily extensible to alkali and alkaline metal ions by modifying the training data in the model. Our current algorithm using CH as the primary measure can be applied to transition metals by swapping the training set. At the same time, the application of our algorithm to alkali and alkaline earth metals also requires the use of carboxyl side chains from Glutamic acid and Aspartic acid (ED) as the primary measure and hydroxyl side chains from Serine and Threonine (ST) as the auxiliary measure besides using the corresponding training set.

## 2. Results

### 2.1 PinMyMetal workflow

**Figure 1:**
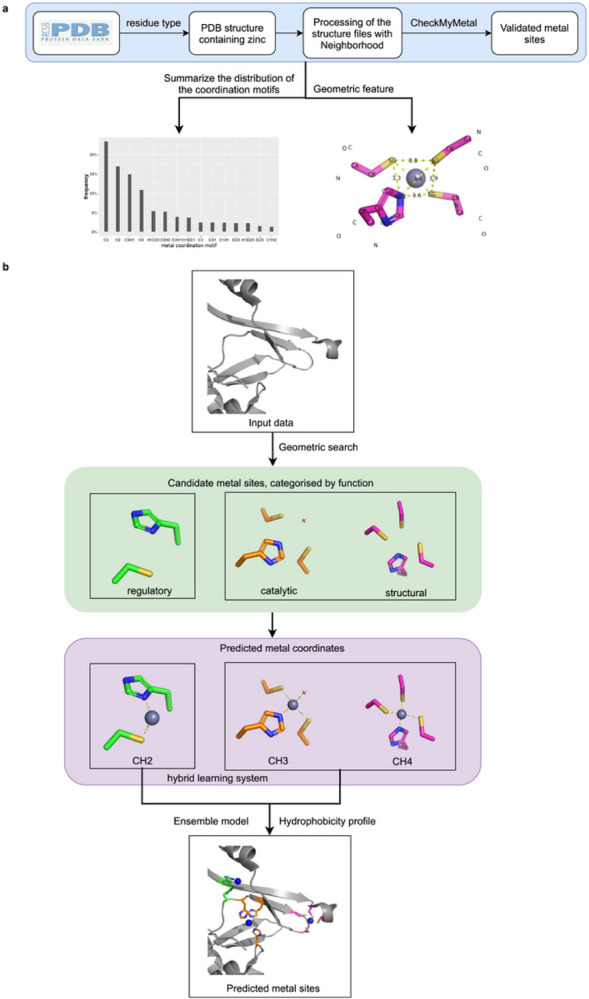
Workflow of PMM. **a**, Obtain validated experimental metal sites and summarize geometric features. **b**, Predict metal binding sites.

The PMM workflow features four modules: (a) CMM validation module; (b) Data analysis and summary module; (c) PMM hybrid learning system; (d) Interactive frontend module. While the latter three modules (b-d) connect sequentially, the validation module interacts with all the other three modules, providing a validated dataset before data analysis to generate a benchmark dataset and confirming the validity of the predicted metal binding site as a utility module (Fig. 1).

Neighborhood processes all zinc-containing protein structure files, followed by validation of zinc binding sites using CMM. PMM is trained using CMM-validated benchmark dataset, utilizing geometric characteristics such as ligand amino acid properties, interatomic distance, angle, and atomic type of the binding site. PMM takes the protein structure as input, searches the entire structure based on ligand type, atomic type, and interatomic distances, and predicts candidate zinc binding sites by constraining geometric features (Fig. 1a).

According to the amino acid atomic coordinates coordinated with zinc, the zinc ion coordinates were deduced. Using the zinc ion coordinates as the center of the sphere, the hydrophilicity profile of atoms within the surrounding 7 Å range was derived (Fig. 1b). Predictors addressing these features are considered for judging the possibility of zinc ions bound to candidate zinc binding sites. The PMM frontend described in more detail in section 2.7 features a web server that allows users to input protein sequence or protein structure to predict zinc ion location and the corresponding coordinated ligands.

### 2.2 The predictive capability and accuracy of the PMM system

PMM first predicts the pair of residues that could potentially bind zinc according to the geometric characteristics from the CMM-validated benchmark dataset, obtaining candidate zinc binding sites. Subsequently, the binding positions of zinc ions are deduced based on the ligand residues of the candidate zinc binding sites. Ultimately, employing a hybrid learning system, further verification is conducted for the candidate zinc binding sites within the CH2 and CH3/CH4 groups using different methods. The CH2 group in zinc binding sites is verified with the ensemble model, while the CH3 and CH4 groups are verified with values of hydrophobicity contrast functions (C) and values of atomic solvation parameters (Δσ) for verification. This is done to determine whether the identified zinc ions truly represent accurate zinc binding sites or are merely false positive hits, lacking evidence to possess zinc binding properties.

**Figure 2:**
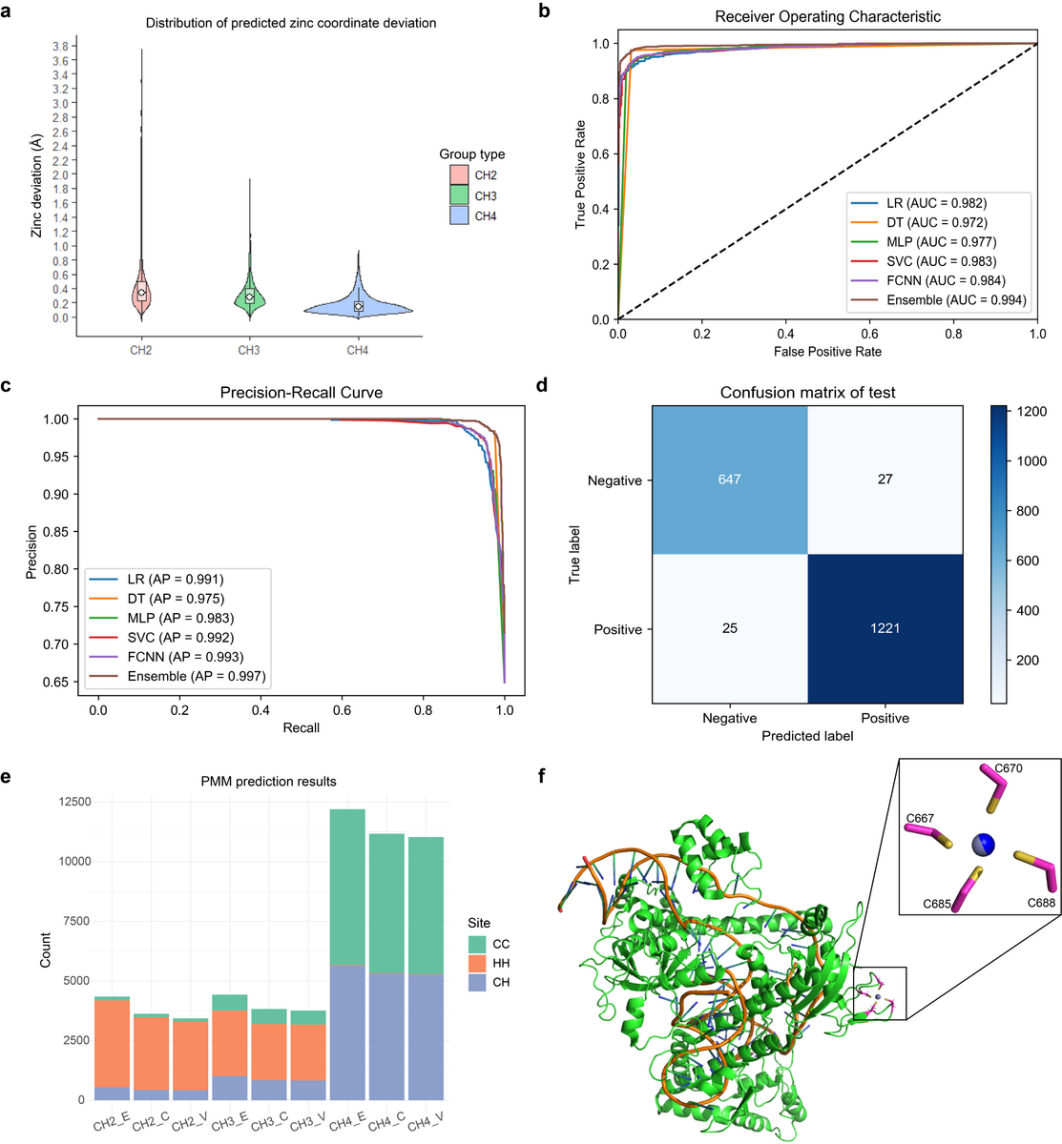
CH2 model Prediction Accuracy Assessment. **a**, Distribution of PMM predicted zinc coordinate deviations for all zinc site groups. For each group, the box plot indicates the median distance deviation (white dot), and the kernel density estimation of all data points is shown as a violin plot with minima and maxima indicated by whiskers. **b**, ROC curves for different models. **c**,P-R curves for different models. **d**, Prediction effect (confusion matrix) of ensemble model in test data. **e**, PMM prediction results for both candidate and verified zinc sites. E: Experimentally determined zinc sites from the CMM-validated benchmark dataset; C: Candidate zinc sites; V: Verified zinc sites. **f**, PMM predicts a single zinc site in the cryo-EM structure 7lyt. The blue spheres represent the predicted zinc site, in agreement with the gray spheres depicting the zinc site modeled by the experimenter.

PMM uses an innovative algorithm to deduce the coordinates of zinc ions. CH2, CH3, and CH4 groups are self-contained in a relatively early classification stage, while each of the CH groups is further divided into six subgroups and uses six subgroup-specific strategies to deduce the most probable location of zinc ions using the known locations of coordinating atoms. These strategies also consider some fundamental measures and complications, including the composition of the coordinating ligands, the orientation of CH sidechain, and possible sidechain rotamer conformations. PMM’s accuracy is evaluated by measuring the distance between the predicted zinc ion location and the experimentally determined location. For CH2, CH3, and CH4 groups, the median zinc deviation is 0.34 Å, 0.27 Å and 0.14 Å, respectively (with average zinc deviation of 0.46 Å, 0.34Å and 0.17 Å, respectively) (Fig. 2a).

An ensemble model is used to verify CH2 candidate zinc binding sites. Receiver operating characteristic curve (ROC curve) and Precision-Recall curve (P-R curve) are employed to assess the prediction performance of different machine learning or deep learning models. Better performance is indicated by convexity towards the upper left corner in the ROC curve and convexity towards the upper right corner in the P-R curve. The area under the ROC curve (AUC) and the area under the P-R curve (AP) are also used as additional measures to assess the prediction performance with an area score range in [0, 1], with higher score indicating better performance. The AUC/AP values of Logistic Regression (LR) model, Decision Tree (DT) model, MLPClassifier (MLP) model, Support Vector Machine (SVC) model, and Fully Connected Neural Network architecture (FCNN) model are 0.982/0.991, 0.972/0.975, 0.977/0.983, 0.983/0.992 and 0.984/0.993, respectively (Fig. 2b, c). The ensemble model exhibits an AUC value of 0.994 and an AP value of 0.997, achieving the highest precision and recall when compared with any of its five base learners. The prediction of the ensemble model in the test set is represented by a confusion matrix (Fig. 2d). According to the confusion matrix, the ensemble model exhibits a recall value of TP / (TP + FN) = 0.981; a precision value of TP / (TP + FP) = 0.978; an F1-score of 2*precision*recall / (precision + recall) = 0.980; and an accuracy of (TP + TN) / (TP + TN + FP + FN) = 0.973.

The prediction of candidate zinc sites is conducted using the CMM-validated benchmark dataset (Supplemental Table 1), and the prediction accuracy is evaluated by IoUR defined in formula (6). For the CH2, CH3, and CH4 group of sites, 3,627, 3,827, and 11,171 sites could be accurately predicted from 4,348, 4,428, and 12,203 experimental sites, indicating a recall rate of 83.4%, 86.4%, and 91.5%, respectively (Fig. 2e). Using IoUR = 1 instead of IoUR ≥ 0.5 as the threshold results in only a slightly reduced number of 3,457 CH2, 3,627 CH3, and 10,466 CH4 group of sites, suggesting a somewhat reduced recall rate of 79.5%, 79.7%, and 85.8%, respectively (Supplemental Table 2). The procedure may exclude some experimental sites from consideration due to certain complications, e.g., for sites with distance between ligands exceeding 4.5Å or for sites with coordinated atoms being N or O of the backbone. Using a hybrid learning system, different strategies are employed for assigning a certainty score to each candidate site within CH2 and CH3/CH4 groups. The certainty score ranges from 0 to 1, and candidates with a score greater than 0.5 are considered verified sites. As a result, PMM recovers 94.7%, 98.3%, and 98.8% verified zinc sites from a total of 3,627 CH2, 3,827 CH3, and 11,171 CH4 candidate zinc sites, respectively (Fig. 2e, Supplemental Table 2). PMM demonstrates high accuracy and recall in predicting the experimental zinc binding sites within the structure. For instance, in the Cryo-EM complex structure of CasPhi-2 (Cas12j) bound to crRNA and Phosphorothioate-DNA (PDB code: 7lyt)^34^, PMM successfully predicted the zinc binding site coordinated by residues C670, C667, C685, and C688 (Fig. 2f), exhibiting a minimal distance deviation of 0.025Å from the zinc site determined by the experimenter.

### 2.3 Prediction of unknown functional sites supported by experimental data

In addition to accurately predicting known experimental binding sites, PMM identifies a large number of previously unknown, putative zinc binding sites, including 2,035 CH2 group, 1,013 CH3 group, and 486 CH4 group of zinc binding sites that are not determined in experimental structures. For these predicted metal binding sites, 425, 98, and 50 sites are from structures determined by cryo-EM, and 445, 304, and 42 sites are metal binding sites that contain another transition metal other than zinc, respectively (Supplemental Table 3).

**Figure 3:**
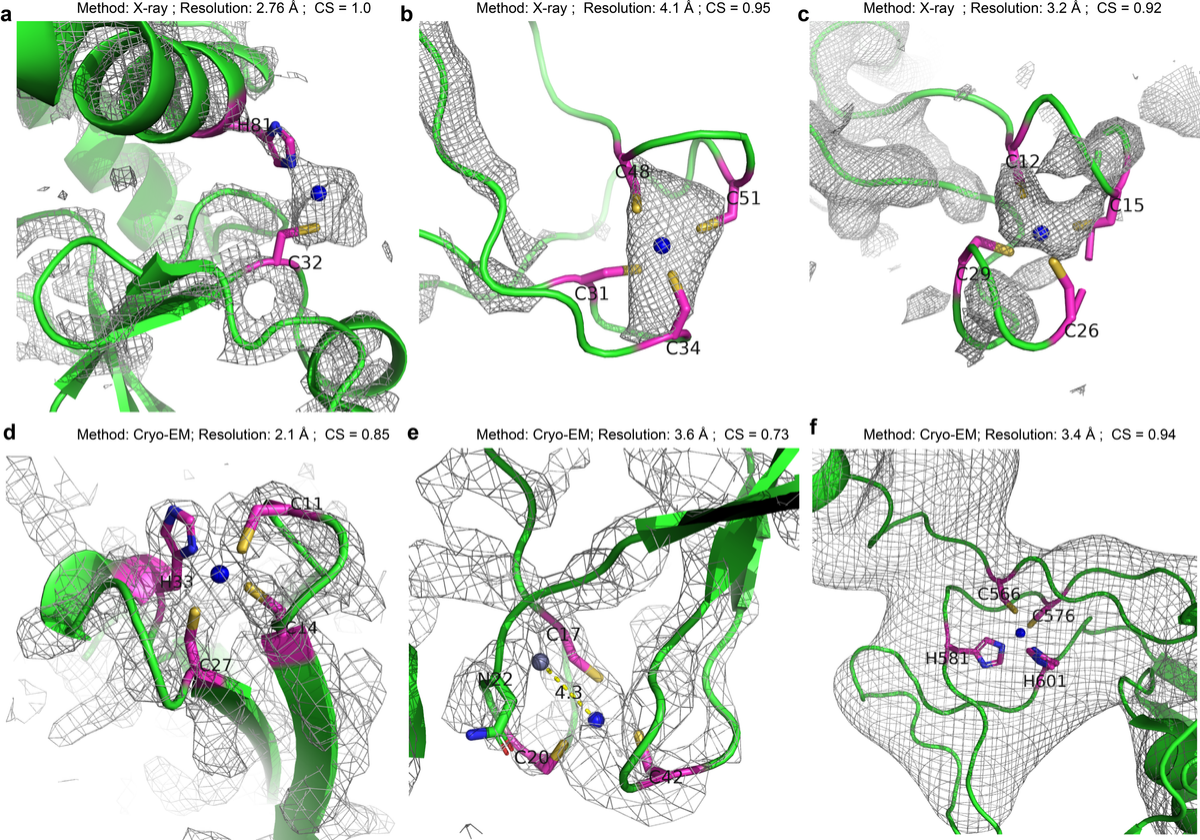
Zinc binding sites predicted by PMM. **a**, 5w8t, chain C, 2-residue zinc site, 2F_o_-F_c_ map with 3.0 σ cutoff. **b**, 1nik, chain L, 4-residue zinc site, 2F_o_-F_c_ map with 3.0 σ cutoff. **c**, 2zp9, chain H, 4-residue zinc sites, 2F_o_-F_c_ map with 1.0 σ cutoff. **d**, 6xz7, chain E, 4-residue zinc site, EM map with 5.0 σ cutoff. **e**, 6exv, chain I, 3-residue zinc site, EM map with 5.0 σ cutoff. **f**, 7pw5, chain B, 4-residue zinc site, EM map with 5.0 σ cutoff. CS: certainty score; Blue spheres represent predicted zinc sites, while gray spheres depict experimentally determined zinc sites; Electron density maps (2F_o_-F_c_ or EM) are shown in gray mesh with optimal σ cutoff in the proximity of the metal sites.

The CH2 group of zinc binding sites is a typical regulatory site that reversibly binds to zinc ions, depending on the zinc concentration or the presence of chaperone protein in the environment. Therefore, the absence of a zinc site under certain experimental conditions does not necessarily exclude its suitability to bind zinc. PMM predicts a zinc binding site in the ORF1ab protein of the MERS-CoV papain-like protease complex with the C-terminal domain of human ISG15 (PDB code: 5w8t)^35^, coordinated by ligands C32 and H81. Although the zinc ion is not determined in the experimental structure, the electron density is observed at the proposed zinc location (Fig. 3a).

CH3 and CH4 groups of zinc binding sites could escape from experimental determination due to several reasons: (1) the similarity of zinc ions with other commonly observed transition metal ions such as Fe, Cu, Mn, etc. can cause promiscuity and thus the presence of ions other than zinc at the predicted zinc location; (2) the experimenter lacks the expertise or accidentally overlooks the modeling of some zinc binding sites during model building; and (3) limited-resolution structures usually exhibit uncertainty in metal ion modeling (Supplemental Table S3). For example, in the low-resolution X-ray structure of wild-type RNA polymerase II (PDB code: 1nik)^36^ determined to a resolution of 4.1 Å, a CH4 site with four cysteine residues does not have the zinc ion modeled despite the presence of electron density (Fig. 3b). Another example is the TRAP-Anti-TRAP complex structure with a resolution of 3.2 Å (PDB code: 2zp9)^37^. On the Tryptophan RNA-binding attenuator protein-inhibitory protein (Anti-TRAP) within this structure, PMM predicted a CH4 site with four cysteine residues. While electron density is observed at this site, it is not modeled in the experimental structure (Fig. 3c).

The number of transition metal ions per 100 amino acids is used as a metric to assess metal annotation efficiency due to the association between lower resolution and higher uncertainty in metal ion modeling. Structures with resolutions better than 2.5Å are excluded due to the scarcity of atomic-resolution cryo-EM structures (41 structures). The cryo-EM method is commonly used for determining large, complex, or challenging-to-crystallize structures. However, the annotation efficiency for transition metal ions is lower in cryo-EM structures compared to X-ray structures of the same resolution range, consistently decreasing from 0.25 metal ions per 100 amino acids at 3Å to 0.05 metal ions per 100 amino acids at 5Å (Fig. S1). PMM is well suited to routinely model missing metal binding sites or annotate candidate metal binding sites in cryo-EM structures. For example, in the structure of the E. coli 50S ribosomal subunit complex with unmodeled metal ions (PDB code: 6xzi)^38^, PMM predicts a zinc binding site on 50s ribosomal protein L36 (Chain e). This site is coordinated by residues C11, C14, C27, and H33, and is supported by an observed peak in the charge density map (Fig. 3d). In the structure of mammalian RNA polymerase II subunit RPB7 (PDB code: 6exv)^39^, PMM predicts a zinc site coordinated by residues C17, C20, and C42. Although this site is not experimentally modeled, it gives an educated estimation of the candidate zinc binding site that is not contradictory to the charge density map. Conversely, a nearby zinc binding site modeled by the experimenter is not reasonably coordinated and lacks experimental support (Fig. 3e). These discrepancies underscore the challenges in cryo-EM structural determination, while PMM’s prediction suggests its potential in supplementing metal binding site modeling. In the structure of the human SMG1-8-9 kinase complex (PDB code: 7pw5)^40^, PMM predicts a zinc binding site of unknown function on the SMG8 protein coordinated by the residues C566, C576, H581, and H601 (Fig. 3f). While the insufficient resolution may not support the direct atomic modeling of metal ions in this model, PMM provides an alternative approach to model coordination bonds pertaining to metal ions in medium-to-low resolution cryo-EM structures.

### 2.4 Comparison with other predictors

The benefits of PMM to other existing predictors (Table 1) can be summarized as follows:

(a) PMM predicts both metal binding residues and metal ion coordinates;

(b) PMM achieves superior prediction accuracy with minimal coordinate error between the coordinates of predicted zinc ions and the actual zinc ions;

(c) PMM is faster than other metal predictors. For a protein consisting of 350 amino acids, the prediction using the PMM online web service takes approximately 15 seconds. When utilizing Metal3D for prediction and relying on local CPU processing, the process takes about 130 seconds. However, when using “Huggingface Spaces” for web-based online prediction, despite not requiring downloads and registrations, Metal3D demands more runtime, taking approximately one day;

(d) PMM embeds validation for all steps from dataset construction to result verification;

(e) PMM specializes in predicting regulatory sites coordinated by only two amino acids besides 3- or 4- 4-coordinated structural and catalytic sites;

(f) PMM employs an objective and thorough search strategy to select a negative dataset, in contrast to ZincBindDB and znMachine, which randomly chose arbitrary sites with reasonable geometry yet no experimental zinc as a negative dataset. This minimizes the inclusion of false negative sites or the exclusion of true negative sites in the negative dataset;

(g) PMM innovatively uses CH as the major criteria and ED as the auxiliary criteria to predict all possible zinc binding sites. The zinc location is used as the center to find amino acids other than CH residues within the range of the first coordination sphere (2.5Å) and the second coordination sphere (4Å).

**Figure 4:**
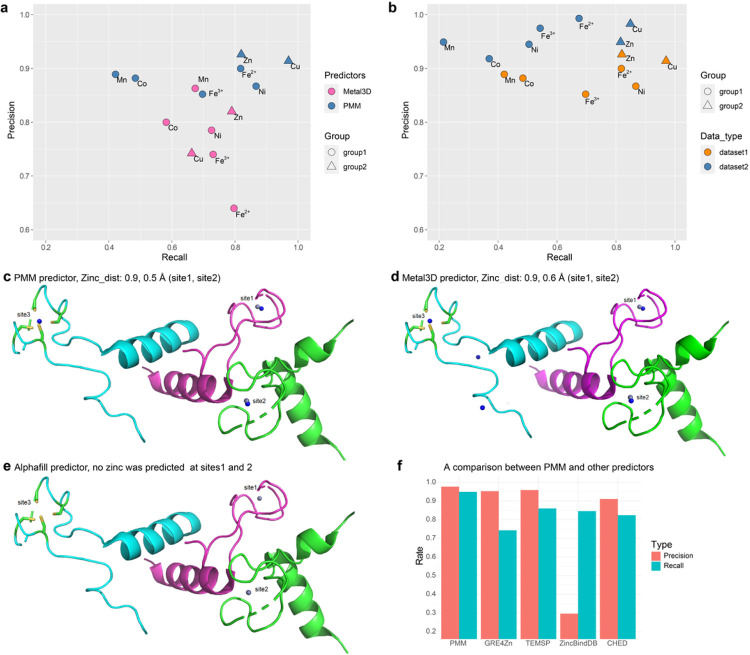
Prediction results of PMM and Metal3D for different transition metals. **a**, Comparison between Metal3D and PMM. **b**, Stability of PMM server using an extended dataset. group1: Mn, Fe, Co, Ni; group2: Cu, Zn; dataset1: other transition metals dataset in Metal3D article; dataset2: CMM-validated dataset with resolution better than 2Å. **(c,d,e)**, Annotation of zinc binding sites in the structure of 2z9p by different predictors. Blue spheres represent predicted zinc sites, while gray spheres depict experimentally determined zinc sites. Only the D, H, and I chains are displayed. **f**, The precision and recall of different predictors on the same dataset.

PMM is compared with representative predictors from each of the three categories with PMM in more detail, including Category I predictors ZincBindDB, znMachine, CHED; Category II predictors Metal3D, AlphaFill; and Category III predictors GRE4Zn, TEMSP. For an apple-to-apple comparison, the same TP and FN definition and the corresponding datasets used in Metal3D and TEMSP are also used to evaluate PMM. When comparing PMM, ZincBindDB, GRE4Zn, TEMSP, and CHED, the evaluation uses a dataset comprising 136 experimentally determined zinc binding sites derived from 100 protein structures^17^. While these data are excluded in the training set of the PMM algorithm to eliminate biases, PMM still identifies 129 out of the total 136 actual zinc binding sites using the same 0.5 IoUR cutoff, achieving a sensitivity/recall value of 94.9%, which notably exceeds the sensitivities predicted by ZincBindDB (84.6%), GRE4Zn (74.3%), TEMSP (86.0%), and CHED (82.4%). PMM also scores a smaller deviation of 0.248 Å when compared with the average deviations of zinc positions predicted by GRE4Zn (0.267 Å) and TEMSP (0.38 Å). Additionally, PMM predicts a precision of 97.7%, outperforming the precision values predicted by ZincBindDB (29.6%), GRE4Zn (95.3%), TEMSP (95.9%) and CHED (91.1%) (Fig. 4f, Supplemental Table 4).

Metal3D is a recently published metal ion position predictor based on 3D convolutional neural networks, which is currently the most accurate metal location predictor with a deviation of 0.70 ± 0.64 Å between predicted positions and experimental locations. PMM features a deviation between predicted positions and experimental locations of 0.323 Å, which is 54% less deviation when compared with Metal3D. The dataset reported in Metal3D includes 189 zinc binding sites from 59 structures and is evaluated using: True Positives (TP) for predictions within 5 Å of an experimental metal site, False Positives (FP) for predictions beyond 5 Å from both actual and other false positive sites, and False Negatives (FN) for experimental sites lacking a predicted metal within the 5 Å threshold. PMM uses the same TP, FP, and FN definition as Metal3D to define a corresponding dataset that contains 205 validated zinc binding sites from the same 59 structures, and achieves a better prediction precision of 0.983, a better recall of 0.571, and a better average zinc-deviation of 0.166Å when compared with the corresponding values from Metal3D (Supplemental Table 5).

In order to compare the selectivity for other common transition metals, a data set of 292 metal binding sites (38 for Mn, 66 for Fe, 31 for Co, 30 for Ni, 66 for Cu, 61 for Zn) is chosen to evaluate both PMM and Metal3D using a precision and recall distribution map. The PMM prediction results are generally better, with a precision that consistently outperforms that in Metal3D (Fig. 4a). Evaluation of average metal deviation indicates that zinc is the metal with the most accurate prediction in both PMM and Metal3D. The average error value of 0.257Å in PMM is better than that (0.52 + 0.45Å) in Metal3D (Supplemental Table 5). An extended CMM-validated dataset with a resolution better than 2Å is used to evaluate the stability of PMM against different data (Fig. 4b). The precision of PMM is consistently increased when using the high-resolution dataset, while the recall values for Mn, Fe, Co, and Ni remain unstable. Trained on zinc, PMM excels in Zn precision and recall, while the similarity between Zn and Cu makes it also a good Cu predictor in terms of both precision and recall. The relatively low recall for Mn, Fe(III), and Co could be attributed to the higher selectivity of the current PMM model trained on the characteristics of Zn data, e.g., towards tetrahedral geometry against octahedral geometry (Fig. 4a,b).

Tools like AlphaFill use structural homology to transplant metals from similar PDB structures to the predicted structure and may not be used to predict novel metal binding sites. For example, PMM predicts a novel metal binding site coordinated by four cysteine residues in a tryptophan RNA-binding attenuator protein-inhibitory protein 2zp9, which is further verified by the presence of electron density map (Fig. 4c,d,e). Since this site was not experimentally observed in either 2zp9 or any other homologous proteins, Alphafill fails to predict its presence (Fig. 4e). Metal3D can predict two zinc locations in this structure with errors of 0.9 Å and 0.6 Å, comparable to the errors in PMM of 0.9 Å and 0.5 Å (Fig. 4c,d). However, Metal3D predicts two additional zinc locations in the same structure, where no electron density is observed, indicating a higher rate of false positive hits of Metal3D when compared with PMM.

### 2.5 Biological implication of zinc binding site prediction for different types of zinc binding sites

**Figure 5:**
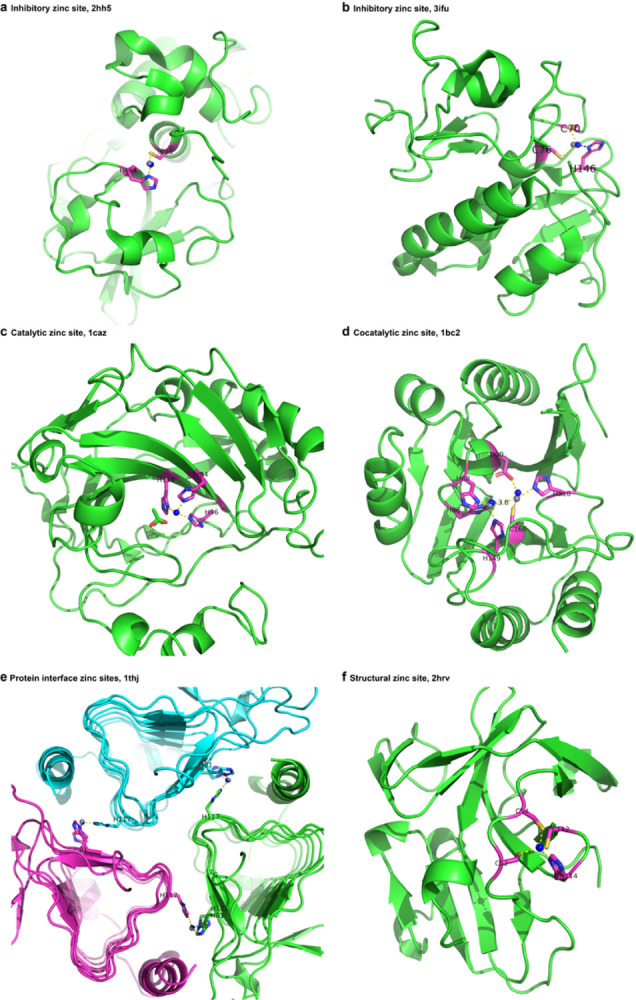
PMM predicts zinc binding sites for different ligands or functions. **a**, Inhibitory zinc site, 2hh5, H172-C273,0.26Å. **b**, Inhibitory zinc site, 3ifu, C70-C76-H146, 0.67Å. **c**, Catalytic zinc site, 1caz, H94_H96_H119, 0.15Å. **d**, Cocatalytic zinc site, 1bc2, Zn1:H88-H86-H149, 0.40Å. Zn2: D90-C168-H210, 0.78Å **e**, Protein interface zinc sites, 1thj, H-81-H117-H122, 0.13/0.10/0.09Å. **f**, Structural zinc site, 2hrv, C52-C54-C112-H114, 0.06Å. The blue spheres represent the predicted zinc site, in agreement with the gray spheres depicting the zinc site modeled by the experimenter.

Although zinc ligands and coordination geometries are largely different among regulatory, catalytic, and structural sites, PMM achieves high accuracy with commendable biological implications in all scenarios (Fig. 5). Zinc ions at the regulatory (inhibitory) and catalytic sites in zinc-containing enzymes require two or three coordinating ligands for full activity (Fig. 5a, b, c). PMM can accurately predict zinc ion location at cocatalytic sites containing two or three metals in close proximity with two of the metals bridged by a side chain moiety of a single amino acid residue, such as Asp, Glu, or His and sometimes a water molecule (Fig. 5d). The application of PMM is not limited to a single polypeptide chain, but also includes protein interface zinc sites formed from ligands supplied from amino acid residues residing in the binding surface of two polypeptide chains (Fig. 5e). Similar to other zinc ions, zinc binding sites on the protein interface can be regulatory, catalytic, or structural.

### 2.6 Open-Source PMM predictor: local and web access

**Figure 6:**
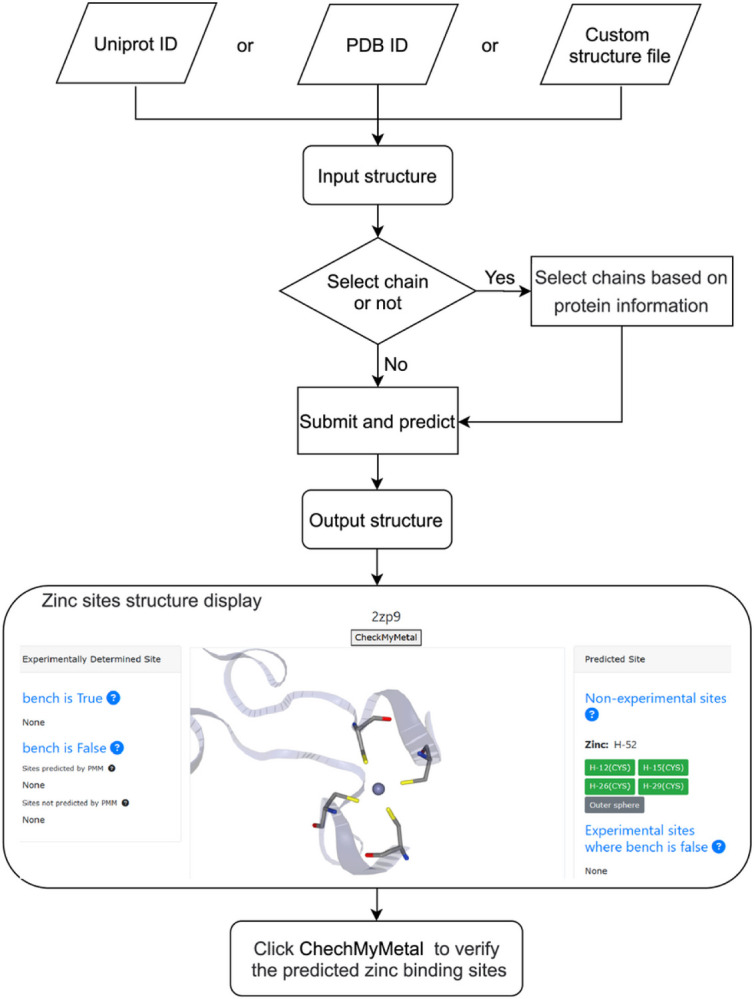
PMM web prediction flow chart.

The code for the PMM predictor is open-source, allowing peers to download, run, and compile it locally. Additionally, an online version is provided for convenient web-based predictions, enhancing the flexibility, ease of use, and user-friendliness in practical applications. The PMM web server is publicly available and freely accessible at https://PMM.biocloud.top. Even though PMM is a structural-based method, it implements an automated structure-retrieval interface that allows users to search by protein name or sequence as identified by Uniprot ID. The server provides three input methods for the acquisition of protein structures for zinc binding site prediction: (1) PDB id from the PDB website; (2) Uniprot ID of the target protein, which will be used to retrieve protein structures from the Uniprot database for further analysis. If multiple experimentally determined structures are found from the same Uniprot entry, structure with the highest sequence completeness and highest resolution is chosen. If no experimentally determined structure is found, a computational model from AlphaFold2 is selected; and (3) PDB or CIF format coordinate file is uploaded by the user (Fig. 6). Pre-processing of the protein structure prompts a chain selection page containing the chain ID, name, source organism, and length for each chain, allowing the user to choose one or more chains of interest to conduct metal binding site prediction.

After submission, users can typically expect to receive a response in about 20 seconds or less. The submitted protein structure, along with all experimental and predicted zinc binding sites, will be displayed on an interactive NGL 3D view page (Fig. 6). The output of PMM is divided into two panels: the right panel features predicted zinc ion location and coordinating amino acid type and residue sequence number (resseq), while the left panel features experimentally determined zinc ion location and coordinating amino acid annotated with whether or not it passes the validation criteria. Experimental zinc binding sites that have not passed the validation criteria is compared with predicted zinc binding sites using IoUR > = 0.5 as the criteria to determine if they are the same site as defined in section 2.2. A “CheckMyMetal” button is provided on the PMM output interface to allow the seamless validation of the predicted zinc binding site on the sister CMM website, with an ‘@’ indicating predicted sites. The experimenter may download the coordinate in PDB or CIF format, with the predicted sites annotated in the ATOM and LINK records. A certainty score between the range of 0 and 1, indicating the confidence value of the zinc binding site, is provided in the occupancy field. The NGL interface also allows the visualization of other non-CH amino acids or small molecule ligands within 4Å of the metal center. Careful examination of the interactions of the zinc coordinating ligands beyond the first coordination sphere could reveal other global characteristics of the protein structure.

## 3. Discussion

PMM adopts CH as the major classification scheme and ED as the auxiliary measure, ensuring sufficient training data for each class of coordination motifs. The biological implications of this classification scheme are validated through the analysis of zinc-containing enzyme structures from the PDB. Considering metal ions in macromolecular structures requires a multidisciplinary approach, coherently considering chemical, crystallographic, biological, and experimental aspects^24^. PMM’s validation procedure, specifically the CMM validation, effectively identifies incorrect metal assignments and suboptimal modeling of metal binding sites. Addressing potential complications, such as geometric distortions of the first coordination sphere, the quality of the diffraction data (e.g., the resolution), and sample preparation concerns, ensures the robustness of PMM in predicting zinc binding sites.

PMM introduces an innovative algorithm that significantly reduces the computational resources required for screening the hydrophobicity contrast function and determining candidate zinc ion locations. By deducing the most probable location before applying the contrast function, PMM maintains accuracy while enhancing efficiency, making it a powerful tool for predicting optimal zinc ion locations within protein structures. Validated zinc ions undergo redundancy removal by measuring the distance between two zinc ions. Two zinc ions would represent the same site if the distances between them are close enough to each other. Compared to Metal3D’s threshold of 5Å for redundancy, we employ a 2.5Å threshold to eliminate redundancy, achieving accurate annotation of binuclear zinc sites while removing redundancy.

As a signal transduction messenger, zinc regulates protein activities, including the inhibition of enzymatic activities, yet this occurs only when the concentration of zinc ions elevates to a certain level. Nevertheless, the inhibitory sites at the active or allosteric sites of enzymes seem to share similar coordination environments with the typical ligand environments of catalytic zinc in zinc metalloenzymes. The only notable distinction is a tendency for lower coordination numbers in regulatory zinc sites. While the K_d_ for zinc ion can range from milli-molar concentration to micro- or nano-molar concentration, how zinc regulates enzyme activity is not clearly defined from the structural perspective. CH2 algorithm provides a one-stop solution to propose a hypothetical mechanism for such inhibition by predicting candidate regulatory (inhibitory) zinc sites and other zinc binding sites coordinated by two CH residues. Many enzyme active sites feature two metal binding amino acid side chains, such as Cys-Cys, His-His, Cys-His, Glu(Asp)-His, and Cys-Glu(Asp), to form a catalytic dyad. Yet not all of them contain two catalytic cysteine or histidine residues, as seen in enzymes like cysteine proteases, protein tyrosine phosphatases (PTPs), aldehyde dehydrogenases, and glyceraldehyde 3-phosphate dehydrogenase^12^. Therefore, failure to predict zinc binding site due to the lack of two CH residues does not necessarily invalidate the possible inhibitory or regulatory role of zinc via an alternative mechanism.

Evaluation of PMM for its selectivity against other transition metals reveals that both Cu and Zn exhibit high precision and recall (Fig. 4a,b). This can be attributed to the general promiscuity of transition metal ion binding according to the Irving-Williams series^41^ with a special characteristic similarity between Cu and Zn (Fig. S2), resulting in the PMM predictor trained on Zn also work with high accuracy for Cu. Most Zn binding sites could also bind Cu in competitive binding conditions, and that selectivity in such cases is not determined solely by the binding site, while the contributions of environmental factors, such as chaperones or compartmentalization, should not be underestimated or overlooked. For the CH4 group of structural sites, it is not uncommon to spot incorrectly assigned zinc ions in metalloprotein structures, especially between Zn and other transition metals. For example, Zn has been assigned as Cu (Fig. S3a, PDB code: 3mnd) or Fe (Fig. S3b, PDB code: 1jyb). However, for CH3 group of catalytic sites and CH2 group of regulatory sites, the border between Zn and other transition metals is rather thin or even overlapping. Therefore, the burden that no algorithm could uniquely determine a specific metal identity among different transition metals for certain metal binding sites stems from the fact that the metal binding site itself is naturally versatile and lacks selectivity, even from a physiological perspective. After all, a protein predicted by PMM to be zinc binding could also bind to multiple metals *in vivo* due to other environmental factors. This also results in the fact that PMM predictor trained on Zn also possesses a relatively high recall rate for most other transition metals besides Zn and Cu (Fig. 4a,b).

In conclusion, PMM can predict metal ion locations and coordinating ligands based on local geometrical and chemical microenvironments. The application of PMM in zinc binding sites exhibits superior accuracy and efficiency performance compared to other predictors, providing a quick way for the scientific community to predict zinc binding sites with easy accessibility, high confidence, and minimal latency. The high efficiency also prompts PMM to excel in the large-scale prediction of metal binding site for the superfamily of metal binding proteins or genomic-scale prediction of metal binding sites. PMM also specializes in predicting regulatory (transient) metal binding sites (2-residue predominate) not specifically handled in any other zinc predictors and exhibits much superior prediction accuracy than Metal3D^23^. Experimentally-determined protein structures generally represent a single snapshot of the protein, while the zinc binding state may not be observed under a specific experimental condition. Therefore, the absence of zinc binding sites in a given crystal structure does not warrant its absence in the associated biological processes. In this sense, PMM opens up a new window of opportunity to examine candidate zinc binding proteins from a perspective not accessible using any known experimental or computational methodologies. We have also demonstrated the effective routine use of PMM to annotate metal binding sites in cryo-EM structures with limited resolution. PMM offers a complementary and accurate solution to model metal ions in cryo-EM structures which would otherwise be challenging due to the limitations of electron penetration depth and scattering effects.

## 4. Methods

### 4.1 Data acquisition, validation, and redundancy elimination

The set of metal-containing protein structures was downloaded using the April 22, 2023 version of the PDB^42^ and processed using the Neighborhood database as described earlier^24^. The intermolecular interaction between metal ions and proteins is stored in the form of coordination bonds and represents the metal binding site. 55,120 experimentally determined zinc ions from 18,082 protein structures were further inspected to remove free zinc ions or zinc ions coordinated by only water, resulting in a dataset of 38,976 zinc binding sites with two or more coordinating ligands from either cysteine or histidine.


(1) $$Q_{e}=\min\left(\left|\frac{\sum V_{i}}{V_{ox}}\right|,\left|\frac{V_{ox}}{\sum V_{i}}\right|\right)$$

(2) $$Q_{c}=1-\frac{\left|v_{1}+v_{2}+\ldots y_{n}\right|}{\sum V_{i}}$$

(3) $$O_{e}=\frac{\sum v_{i}O_{i}}{\sum v_{i}}$$

(4) $$B_{e}=\frac{\sum vB_{i}}{\sum v_{i}}$$

(5) Qe=min(2×min(Om,Oe),1)×min(BmOmBeOe,BeOeBmOm)

The quality of zinc binding site is evaluated using CheckMyMetal (CMM)^32^, with modification based on the previously described algorithm used to validate magnesium binding sites in nucleic acid structures^43^. Since the previous algorithm was tested for magnesium ions, the validation parameters are adapted to be applicable to other metal binding sites. Three parameters were used to quantitatively evaluate the agreement with expected valence (oxidation state) (Q_v_)^(2)^, completeness of the first coordination sphere (Q_c_)^(3)^, and experimental agreement (B factor and occupancy) with the environment (Q_e_)^(6)^. In all formulas, *v*_*i*_ represents the bond valence vector of coordination bond *i*. In formulas (1)–(3), *V*_*i*_ represents the magnitude of bond valence vector *v*_*i*_; *V*_*ox*_ represents the expected oxidation state. In formulas (4)–(5), *B*_*m*_ and *B*_*e*_ represent the B factor of metal (m) or environment (e); while *O*_*m*_ and *O*_*e*_ represent occupancy of metal (m) or environment (e). Each of the three validation parameters Q_c_, Q_v_, and Q_e_ has a valid range of 0 and 1, with 1 indicating the best quality and 0 indicating the worst quality.

The validation procedure is fine-tuned based on the number of coordinating ligands, assuming that four ligands comprise a stable zinc coordination sphere that adopts a tetrahedral coordination geometry^44^. For zinc with 3 or 4 coordinating ligands, a threshold of half of the optimal quality was set as the validation criteria: Q_v_ > 0.5 and Q_c_ > 0.5 and Q_e_ > 0.5. For zinc with two coordinating ligands, while the expected oxidation state *V*_*ox*_ stays at 2, the optimal theoretical bond valence summation (∑*V*_*i*_) is 1, and the optimal theoretical vector sum is |v1 + v2|=0.58. Therefore, the optimal Q_v_ would be 0.5 according to formula (1), and the optimal Q_c_ would be 0.71 according to formula (2). Using a threshold of half of the optimal quality would result in different validation criteria: Q_v_ > 0.25, Q_c_ > 0.355 and Q_e_ > 0.5. Structures containing zinc binding sites passing our validation criteria are subject to clustering using CD-Hit^45^ at 30% sequence identity cutoff to determine homologous zinc binding sites. For clusters containing more than one zinc binding site, the site with the best quality is chosen as the representative zinc binding site for further analysis. A CMM-validated benchmark dataset was ultimately obtained, comprising 15,353 non-redundant structures and 20,979 zinc binding sites. This benchmark dataset is used to train PMM (Supplemental Table S1).

### 4.2 Classification of metal binding sites

CHED residues (Cysteine, Histidine, Glutamic acid, Aspartic acid) are the most common coordinating residues or metal ions, while the use of donor atoms of other amino acids, such as serine, threonine, or lysine, is rare and accounts for less than 1% of all cases of metal-ligand interactions^46^. Hard and soft acids and bases imply that zinc proteins containing sulfur and nitrogen donors in the coordination sphere are more stable than those containing oxygen donors^25, 26^, which also applies to other transition metals, including Mn, Fe, Co, Ni, and Cu. Coordinating ligand analysis of the high-quality non-redundant dataset also reveals that cysteine and histidine are the major contributors to zinc binding sites, with 34,536 zinc ions coordinated by two or more CH residues (85.9%) and 5,690 zinc ions coordinated by zero or one CH residues together with ED residues (14.1%) (Fig. S2). While copper exhibits a similar preference towards CH residues as zinc, the other commonly-observed transition metals exhibit a preference towards HED residues, except for iron-sulfur clusters (Fig. S2). Moreover, while Cu and Zn are coordinated predominately by tetrahedral geometry, Mn, Fe, Co, Ni take both octahedral and tetrahedral geometries.

To reduce the number of classes and ensure sufficient training data for each class of coordination motifs, PMM uses CH as the major classification scheme and ED as the auxiliary measure. This metal ion classification approach fundamentally differs from the principles used in existing metal coordination motif classifiers such as ZincBindDB^19^. ZincBindDB considers all CHED combinations and is only able to predict sites with a sufficient number of cases, such as the top 10 most populated classes (C2H1, C2H2, C3, C3H1, C4, D1H1, D1H2, E1H1, E1H2, H3). For CHED combinations with less experimentally determined structures, ZincBindDB is either unable to build a prediction model, or the prediction accuracy would be seriously compromised. PMM formulates a straightforward classification scheme using the total number of cysteine and histidine as the major criteria. Different combinations of CH are considered as separate classes of coordination motifs. The scheme can accommodate any CH combinations with a sufficient number of training cases, ensuring higher prediction accuracy. According to this criterion, the CMM-validated benchmark dataset is divided into 4,348 CH2 (2-residue, CC, CH or HH) group sites from 3,936 structures, 4,428 CH3 (3-residue, CCC, CCH, CHH or HHH) group sites from 4,041 structures, and 12,203 CH4 (4-residue, CCCC, CCCH, CCHH, CHHH or HHHH) group sites from 7,376 structures (Supplemental Table S1). PMM does not overlook the auxiliary measure of ED residues but rather postpones its consideration after the location of the zinc ion is determined. For example, the structure metallopeptidase (PDB code: 2qvp) contains a zinc binding site B460 coordinated by 2 histidine residues, while a third and fourth coordinating ligands Glu and water is also identified after the location of the zinc ion is predicted (Fig. S4).

The validity of CH classification scheme is further verified by its biological implications. Zinc is a ubiquitous cofactor for all six major classes of enzymes and zinc-containing enzyme structures from the PDB are analyzed. Sites from CH4 group lack catalytic capability and are considered as structural sites, featuring cysteine as the most prominent coordinating ligand, followed by histidine, with the most common combinations being C4 and C3H1. Zinc may contribute to the catalytic activity in sites from CH3 or CH2 group, featuring histidine as the most prominent coordinating ligand, followed by cysteine, with many common CH combinations in different scenarios (Supplemental Table S6). Catalytic zinc generally forms complexes with any three nitrogen, oxygen, and sulfur donors from CHED residues, with histidine (usually the Nε2 nitrogen) being the predominant amino acid because of its capacity to disperse charge through H-bonding of the other non-liganding nitrogen (usually the Nδ1 nitrogen)^14^.

### 4.3 Prediction of candidate zinc binding sites

According to the geometric characteristics of zinc binding sites in the CMM-validated benchmark dataset, PMM searching throughout the protein structure to identify candidate zinc-binding sites based on ligand type, quantity, coordination atom types, and interatomic distances (Fig. S5). The specific geometric restrictions are as follows:

Zinc-coordinating atoms are limited to SG from cysteine and ND1, NE2, CE1, or CD2 for histidine. The delta and epsilon carbon atoms from the histidine side chain are also included due to the possible presence of alternative conformation or mislabeling^47^. The presence of proximal SG atoms from cysteine side chains may implicate the presence of either zinc binding sites or disulfide bonds, depending on the distances between SG atoms. A survey of the distance between SG atoms in protein structures reveals the presence of two peaks, with the smaller peak below 2.2 Å indicating a disulfide bond and the larger peak above 2.8 Å indicating metal binding sites (Fig. S6). The disulfide bond peak (μ = 2.058 Å, σ = 0.133) is excluded using a p-value cutoff of 0.01, corresponding to a Z value of 2.575. The upper limit of the confidence interval is determined as μ_0_ = 2.058 Å +0.133 Å *2.575 = 2.400 Å using two tail t-test, and therefore, pairs of cysteine residues with a distance of SG atoms below 2.4 Å are excluded from further analysis. However, if the distance is too far, the interactions are weaker, affecting the stability of the binding site. Therefore, the interatomic distance is restricted to the range of 2.4 to 4.5 Å.

The datasets of candidate zinc binding sites complying with all criteria with 2-residue, 3-residue, and 4-residue coordinating ligands are individually identified and combined, followed by the removal of redundant zinc binding sites. Two predicted zinc ions are considered redundant if they are too close to each other to form a dinuclear site. Investigation of zinc ion distance distribution reveals that the majority of the distance is between 3Å and 4Å, representing the presence of dinuclear zinc binding sites (Fig. S7a). While Metal3D uses 5Å to determine the presence of occupancy redundancy, we disagree with their threshold since the dinuclear zinc binding site would be mislabeled in Metal3D (Fig. S7b). PMM adopts a threshold of 2.5Å to eliminate the occupancy redundancy yet retains the capability to annotate dinuclear zinc binding sites accurately (Fig. S7a). The accuracy of predictions is measured using the ‘intersection over union ratio’ (IoUR), which quantifies the accuracy of results by balancing the numbers of correctly and wrongly predicted ligand residues for a specific binding site. While IoUR = 1 when the predicted ligands precisely match the actual ligands, we use a threshold of IoUR ≥ 0.5 to indicate true positive (TP) hits ^(6)^.


(6) $$\text{ IoUR }=\frac{\text{N (predictedligandresidues }\cap \text{ actualligandresidues })}{\text{N (predictedligandresidues }\cup \text{ actualligandresidues })}$$

### 4.4 Determination of zinc ion location

The determination of optimal zinc ion location has been computationally intensive before the development of PMM. Characterizing zinc binding sites involves assessing features such as the ‘hydrophobicity contrast function,’ which quantifies the hydrophobicity difference between outer and inner atoms in a stabilizing shell. Metal binding sites exhibit higher hydrophobicity contrast values, with the metal center coordinated by a hydrophilic atomic group shell (containing oxygen, nitrogen, or sulfur atoms) embedded within a larger hydrophobic atomic group shell (containing carbon atoms)^48^. This qualitative observation can be described analytically by the hydrophobicity contrast function C, which is evaluated from the structure and characteristics of different types of metal ions. However, screening the hydrophobicity contrast function at dense grid points to determine candidate zinc ion location in the protein structure requires much computational resources.

PMM uses an innovative algorithm to deduce the most probable location of zinc ions prior to the application of the hydrophobicity contrast function, greatly reducing the number of evaluations needed without compromising the accuracy. Different strategies are adopted to deduce the location of the zinc ion based on the number of coordinating CH residues being CH2, CH3, or CH4 group. While CH2 group is further divided into CC, CH, and HH subgroups (subgroups a-c), CH3 group is further divided into HHH subgroup and other CH3 subgroup (subgroups d-e). With CH4 group having a single handling procedure (group f), a total of six strategies used to cover all scenarios are described in more detail below.

**Figure 7:**
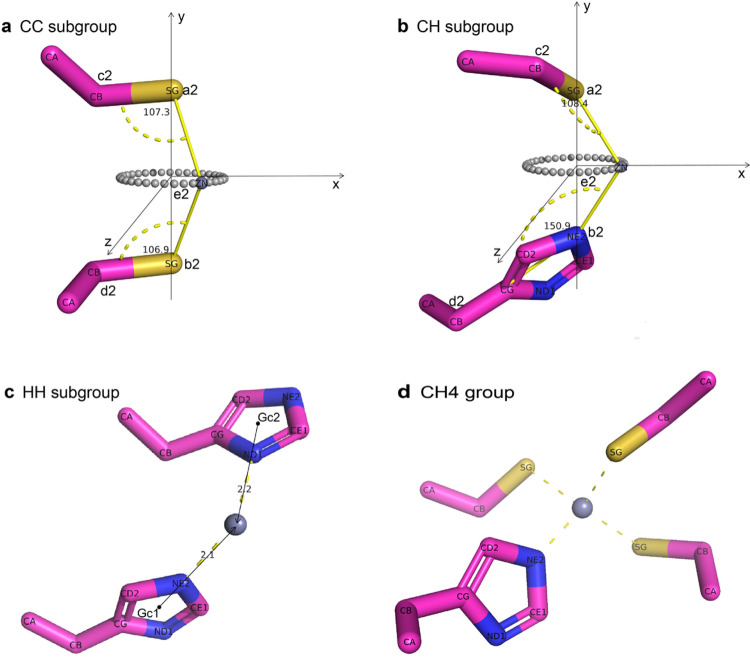
Schematic diagram of zinc ion coordinate prediction algorithm. **a**, CC subgroup. **b**, CH subgroup. **c**, HH subgroup. **d**, CH4 group.

(a) CC subgroup: A segment a_2_b_2_ is drawn between the two Sγ atoms with the coordinate of the midpoint marked as e_2_, which is also on a plane p_2_ perpendicular to the segment a_2_b_2_ (Fig, 7a). The theoretical distance between zinc ion and e_2_ of 1.2Å is deduced based on the average distance between coordinating ligands being 3.6 Å (Fig. S5), and the average coordinating bond distance of 2.1 Å. The optimal location of zinc ion is restricted on the plane p_2_ and has a theoretical distance of 1.2Å from e_2_, resulting in a collection of points forming a circle. The two Sγ atoms coordinating the zinc ion should feature a Zn-Sγ-Cβ angle of 109° and be on the distal side of Cβ to dodge possible clash (Fig. 7a). A scoring function is used to evaluate the deviation from a Zn-Sγ-Cβ angle of 109° for each point from the abovementioned circle. The highest-scored point is chosen as the optimal location of the target zinc ion.

(b) CH subgroup: While coordinating cysteine features a Zn-Sγ-Cβ angle of 109°, statistical analysis reveals that coordinating histidine features a Zn-Nδ1-Cβ angle of 100° and a Zn-Nδ2-Cβ angle of 155° (Fig. S8). The strategy a for CC subgroup is slightly modified to accommodate this difference (Fig. 8b).

(c) HH subgroup: The gravity centers G_c1_ and G_c2_ are calculated using the five atoms forming the corresponding five-member ring. All four atoms Cδ2, Nε2, Cε1, Nδ1 on the five-member ring of the histidine sidechain are considered as candidate coordinating atoms. Four rays G_c1_-Cδ2, G_c1_-Nε2, G_c1_-Cε1, G_c1_-Nδ1 are drawn for the first five-member ring, with 2.1Å segments G_c1_z_1_, G_c1_z_2_, G_c1_z_3_, G_c1_z_4_ aligned with each ray, and z_1_, z_2_, z_3_, z_4_ being the candidate zinc location, respectively. The candidate zinc location for the second five-member ring is deduced using the same procedure and denoted as y_1_, y_2_, y_3_, y_4_. The distance between each candidate zinc location from z_1_, z_2_, z_3_, z_4_ and each candidate zinc location from y_1_, y_2_, y_3_, y_4_ are calculated to determine the closest pair of candidate zinc ions (Fig. 7c). The average coordinate of this pair is chosen as the optimal zinc location.

(d) HHH subgroup: Three candidate zinc locations are deduced using strategy c for HH subgroup. The average coordinate of these three locations is chosen as the optimal zinc location.

(e) Other CH3 subgroup: Three candidate zinc locations are deduced using the strategies a-c for CC, CH, and HH subgroups. A voting mechanism is implemented in this scenario since cysteine is more liable to adopt a conformation not suitable to coordinate metal when compared to histidine. Three distances are calculated from each pair of candidate zinc locations, with the shortest distance considered a major vote (2 out of 3). The average coordinate of these two candidate zinc locations is chosen as the optimal zinc location.

(f) CH4 group: The center of the four zinc-coordinating atoms is chosen as the optimal location of a potential zinc ion (Fig. 7d).

### 4.5 Calculation of hydrophobic profiles

The zinc ion location is used as the center of the sphere to calculate the hydrophobicity contrast functions values (C) and mean atomic salvation parameters values (Δσ)^48^. For each identified zinc ion location, a series of 21 radii ranging from 2 Å to 7 Å, with a step size of 0.25 Å (2, 2.25, 2.5, …, 7), are chosen to generate hydrophobicity contrast curves (Fig. S9a, c) and mean atomic solvation parameter curves (Fig. S9 b, d). The hydrophobic profiles are used not only in calculating certainty score for each predicted zinc ion, but also as parameters in the ensemble model.

### 4.6 Verification of candidate zinc binding sites

Predicted candidate zinc sites are subject to different verification strategies according to CH2 versus CH3/CH4 groups. For zinc binding sites from the CH2 group, the structural characteristics of each ligand residue and the hydrophilic characteristics of amino acids within a radius of 7 Å from zinc ions are used to construct an ensemble model for further verification of the candidate zinc sites. For zinc binding sites from the CH3/CH4 groups, the Pearson correlation coefficient is used to evaluate the similarity in hydrophilic characteristics between the predicted and experimental binding sites, contributing to the further verification of the candidate zinc sites. Our strategies and verification methods for distinct sites are referred to as a hybrid learning system.

The prediction of CH3 and CH4 groups of zinc binding sites is generally straightforward since most zinc ions adopt a typical tetrahedral conformation. We use the proximal interaction network of 3 or 4 CH residues as a strong signal to procure a candidate list of zinc binding sites. The prediction accuracy can easily achieve 85% or higher with geometric restriction of amino acid type, atom type, and coordination bond distance (Supplemental Table S2). The hydrophobicity profile is used for further processing and evaluation, analyzing the values of hydrophobicity contrast functions (C) and atomic solvation parameters (Δσ) (Fig. S9c, d). The certainty score of the predicted site is determined by calculating the Pearson correlation coefficient between the C values and Δσ values curves of the predicted site and the corresponding curves obtained from the experimental site. A certainty score higher than 0.5 is used as the criterion to further verify the identity of the zinc binding site. The calculated certainty score is annotated in the occupancy field of each atom record for zinc ion in the output coordinate file.

The prediction of CH2 group of zinc binding sites require the use of a sophisticated ensemble model to achieve the optimal prediction accuracy. Predictors used in the ensemble model can generally be categorized as ligand type, geometrical parameters, and hydrophobic profiles (Supplemental Table S7). Ligand types including coordinating amino acid residue names (C or H) and coordinating atom names (Sγ, Cδ2, Nε2, Cε1, Nδ1) are enumerated using One-Hot Encoding. Geometrical parameters are numeric values including coordinating atom distance, Cα distance, Cβ distance, and four angels representing the relative positions and orientations of the C_α_ and C_β_ atoms. Hydrophobic profiles feature 21 hydrophobicity contrast function values (C) and 21 mean atomic salvation parameters values (Δσ). A compilation of the three categories of data result in a total of 61 predictors used for further model training.

After excluding multi-conformational sites, a total of 4,151 experimentally determined sites from the CH2 group are used as positive datasets, including 134 CC, 3,495 HH, and 570 CH sites. To obtain a negative dataset, the potential zinc binding sites predicted in the first step are screened for the absence of another metal ion within 4 Å of the site and conform to the criteria of either Qc < 0.355 or Qv < 0.25. A total of 2,246 sites from the CH2 group that fail one of the validation criteria are used as the negative dataset, including 108 CC, 1,543 HH, and 547 CH sites. The data are stratified according to the CH group and split with 70% of the data as the training set and the remaining 30% as the test set to evaluate the effect of the classification model (Supplemental Table 8). An ensemble model is carried out with five Base Learners encompassing both machine learning and deep learning learners to prevent potential underfitting or overfitting due to the use of a single algorithm. The four machine learners include LR, DT, MLP, and SVC, while the deep learner is a FCNN architecture implemented with the Keras library. Individual predictors using the five different algorithms are trained with 10x cross-validation to pick the optimal parameter. Results of the five base learners are combined to form a strong learner ensemble model based on a major voting method (3 + out of 5) using a homemade script. The ensemble model performs classification to distinguish between zinc and non-zinc binding sites and outputs a probability value for each site as a certainty score. The calculated certainty score based on the hydrophobic profile is then annotated in the occupancy field of each atom record for zinc ion in the output coordinate file.

### 4.7 Web service implementation

PMM web server is deployed using an Ubuntu Linux virtual machine running Nginx 1.14.0 and Gunicorn 20.0.4. The interface components of the website are designed and implemented using the Django template engine 3.1.4. Molecular graphics on the view page use HTML5 as implemented in the NGL Javascript library. PMM has been tested in several popular web browsers, including Google Chrome 89.0.4389.82, Mozilla Firefox 87.0, Apple Safari 13.0.2 and Microsoft Edge 89.0.774.75. The styles of the web interface are optimized using the Bootstrap 4.5.0 library to accommodate both large computer screens and small screens on handheld devices. The PMM webserver is accessible via https://PMM.biocloud.top.

## Figures and Tables

**Table 1:** Comparison with other metal predictors

Predictor	Category	Input data	Method	Output data	Type and number of ligands	Provide zinc ion location	Provide a structural model	Typical response time	Year of publication
PMM	III	Structure, Uniprot ID	Geometry, ML	PDB file, Structure	CH ≥ 2	Yes	Yes	5–50 seconds	2023
Metal3D	II	Structure	CNN	Zinc ion location	N/A	Yes	No	3–60 minutes	2023
AlphaFill	II	Structure, Uniprot ID	Structure homology	PDB file, Structure	N/A	Yes	Yes	5–50 seconds	2023
ZincBindDB	I	Structure, Sequence	ML	Predicted sites	CHED ≥ 2	No	No	3–10 minutes	2021
znMachine	I	Sequence	ML	Predicted sites	CHED ≥ 3	No	No	unavailable	2019
GRE4Zn	III	Structure	Geometric REstriction	PDB file	CHED ≥ 3	Yes	No	5–30 seconds	2014
TEMSP	III	Structure	ML	PDB file	CHED ≥ 3	Yes	No	unavailable	2011
CHED	I	Structure	ML	Predicted sites	CHED	No	Yes	unavailable	2007

## Data Availability

The data used to train and test the model and other source data has been deposited in Figshare under accession code (https://doi.org/10.6084/m9.figshare.25011212).
